# The opposing action of stromal cell proenkephalin and stem cell transcription factors in prostate cancer differentiation

**DOI:** 10.1186/s12885-021-09090-y

**Published:** 2021-12-15

**Authors:** Alvin Y. Liu

**Affiliations:** Department of Urology and the Institute for Stem Cell and Regenerative Medicine, University of Washington, Seattle, WA 98195 USA

**Keywords:** Prostate cancer differentiation, Stromal factor PENK, Stem cell factors, Luminal-like adenocarcinoma, Stem-like small cell carcinoma, Differentiation marker AGR2

## Abstract

**Background:**

Loss of prostate cancer differentiation or de-differentiation leads to an untreatable disease. Patient survival would benefit if this can be prevented or reversed. Cancer de-differentiation transforms luminal-like (differentiated) adenocarcinoma into less luminal-like and more stem-like (undifferentiated) small cell carcinoma through a sequential activation of stem cell transcription factors (scTF) POU5F1, LIN28A, SOX2 and NANOG. Like stem cells, prostate small cell carcinoma express this quartet of scTF as well as a 10-fold lower level of β2-microglobulin (B2M) than that of differentiated cell types. In organ development, prostate stromal mesenchyme cells mediate epithelial differentiation in part by secreted factors.

**Methods:**

The identified prostate stromal-specific factor proenkephalin (PENK) was cloned, and transfected into scTF^+^B2M^lo^ stem-like small cell carcinoma LuCaP 145.1, reprogrammed luminal-like scTF^−^B2M^hi^ LNCaP, and luminal-like scTF^−^B2M^hi^ adenocarcinoma LuCaP 70CR. The expression of scTF, B2M and anterior gradient 2 (AGR2) was analyzed in the transfected cells.

**Results:**

PENK caused down-regulation of scTF and up-regulation of B2M to indicate differentiation. When transfected into reprogrammed LNCaP, PENK reversed the reprogramming by down-regulation of scTF with attendant changes in cell appearance and colony morphology. When transfected into LuCaP 70CR, PENK up-regulated the expression of adenocarcinoma antigen AGR2, a marker associated with cancer cell differentiation.

**Conclusions:**

Prostate cancer cells appear to retain their responsiveness to stromal PENK signaling. PENK can induce differentiation to counter de-differentiation caused by scTF activation. The many mutations and aneuploidy characteristic of cancer cells appear not to hinder these two processes. Loss of prostate cancer differentiation is like reprogramming from luminal-like to stem-like.

**Supplementary Information:**

The online version contains supplementary material available at 10.1186/s12885-021-09090-y.

## Background

Development of the urogenital organs prostate and bladder involves intercellular signaling in differentiation [1]. This cell-to-cell communication is controlled by secreted hormone molecules of stromal mesenchyme cells and heterotypic cell contact. Prostate stromal proenkephalin (PENK) was identified by a comparative transcriptome analysis between sorted CD49a^+^ prostate stromal cells and CD13^+^ bladder stromal cells of the lamina propria [2], the rationale being that genes encoding these molecules are likely organ specific (i.e., either prostate or bladder). From this analysis, 288 prostate and 91 bladder stromal genes were identified. Organ-specific expression was validated by reverse-transcriptase polymerase chain reaction (RT-PCR) analysis [2]. Genes encoding secreted proteins were identified by the presence of *N*-terminal signal peptide sequences. PENK was the lead differentially expressed gene in prostate. Immunostaining with a PENK polyclonal antibody raised against the peptide sequence T163-E179 detected strong reactivity in the stroma of benign prostate tissue and no reactivity in the bladder lamina propria [2]. This result validated the RNA expression analysis. Parenthetically, prostate PENK was not processed to enkephalin opioids as indicated by the immunostaining pattern of enkephalin antibodies reported in the literature [3]. Prostate stromal cells are not known as a source of these opioids.

Defects in stromal signaling could be the basis for diseases of the prostate such as hyperplasia and neoplasia. One defect could be the absent production of key signaling molecules. The cancer-associated stroma of prostate tumors was found to have no expression of PENK as found by immunostaining, expression analysis of microdissected tumor tissue samples [2], and dataset query of sorted CD90^+^ cancer-associated stromal cell transcriptomes [4]. Absent immunostaining in the tumor stroma also indicated no appreciable diffusion of the secreted PENK produced from neighboring benign stroma [5]. Cancer cells of tumor glands are separated by about twenty CD90^+^ cancer-associated stromal cell width from benign tissue CD49^+^ stromal cells [6]. This suggests that tumor cells are not signaled by PENK from benign tissue.

As a functional test of stromal cells, an embryonal carcinoma (EC) cell line, NCCIT, was cultured in stromal cell conditioned media [7]. NCCIT was established from a germ cell tumor [8], and has a near identical transcriptome as that of embryonic stem (ES) cells [9, 10]. Our experimental results showed that factors in the prostate stromal cell media could induce NCCIT to differentiate into stromal-like cells in a time course of 7 d. Differentiation was evidenced by changes in transcriptome with duration of culture as well as cellular and colony morphology [7]. Like stem cells, NCCIT exhibited plasticity in response where gene expression induction was different by conditioned media of bladder stromal cells [7], or prostate cancer-associated stromal cells [11]. PENK was specifically induced by prostate stromal cell media but not by the other stromal conditioned media.

Multiple prostate cancer cell types have been characterized, from adenocarcinoma, non-adenocarcinoma to small cell carcinoma. Based on transcriptomes, these various types could be clustered in two groupings with respect to the differentiated prostate cell types of CD26^+^ luminal, CD104^+^ basal, CD49a^+^ stromal, plus CD31^+^ endothelial, and ES cells [9]. They are either luminal-like (i.e., proximal to luminal cells) or less luminal-like, more stem-like (i.e., distal to luminal cells but proximal to stem cells). Cancer cells of adenocarcinoma with glandular differentiation are luminal-like. Cancer cells of non-adenocarcinoma and small cell carcinoma without glandular differentiation are more stem-like. These different types are represented by a family of over 40 LuCaP patient-derived xenograft (PDX) lines [12]. Transcriptome of the stem-like small cell carcinoma LuCaP 145.1 is similar to that of stem cells [13]. Of note, LuCaP 145.1 expresses the stem cell transcription factors (scTF) LIN28A, NANOG, POU5F1, SOX2, as well as a low level, compared to that of differentiated cells, of β2-microglobulin (B2M) [14]. Thus, LuCaP 145.1 displays a stem cell phenotype of scTF^+^B2M^lo^. In contrast, adenocarcinoma LuCaP lines are generally negative for scTF except POU5F1, and are B2M^hi^ [13, 14]. Transfection of this quartet of scTF converted adenocarcinoma LuCaP 70CR, 73CR, 86.2, 92, 105CR (CR = castration resistant) into stem-, small cell carcinoma-like derivatives with changes in transcriptome and cell morphology [13] as in reprogramming. The four scTF cDNA cloned from LuCaP 145.1 could in turn reprogram normal fibroblasts as well as prostate cancer cells [14].

The experiments reported here were designed to show that prostate cancer cells, like normal prostate cells, might also respond to stromal PENK. First, full-length PENK cDNA was cloned from prostate tissue, and transfected into LuCaP 145.1 using a plasmid vector. Transfection ensured that PENK was the responsible factor. For transfection, the in vivo-passaged LuCaP 145.1 cells were adapted to grow on irradiated mouse embryonic fibroblasts (MEF). Second, if PENK could affect stem-like cancer cells it might also reverse reprogramming of scTF-transfected luminal-like cancer cells. Third, not only stem-like cancer cells but also luminal-like cancer cells could respond to PENK signaling. Adenocarcinoma LuCaP 70CR, which was obtained from passages of LuCaP 70 in castrated animals [12], was used as an example. The absence of androgen stimulation as in a castrated host affects cell differentiation through the activity of androgen receptor (AR). Expression of the differentiation-associated adenocarcinoma antigen anterior gradient 2 (AGR2) [15] was examined. Clinical utility of this research lies in the potential application of differentiation therapy to treat prostate cancer.

## Methods

### Mouse embryonic fibroblast (MEF) feeder

Preparation of MEF from mouse embryos was previously described [13]. The harvested cells were expanded in RPMI1640 media supplemented with 10% FBS, and frozen for storage in N_2_ at low passage. Stocks were thawed and expanded. Near confluent cells from a dozen 10-cm plates were resuspended in 5 ml media, and irradiated at 3000 rad for 5 min. The treated cells were cultured on 0.1% gelatin-coated plates 1d prior to plating with LuCaP cells, or frozen for later use.

### Culture of LuCaP cells on MEF

LuCaP cancer cells were harvested from animal hosts (Fox Chase C.B-17 SCID male mice, Charles River Laboratories, Wilmington, MA). Procedures used in tumor harvest were reviewed and accepted by UW animal welfare. This study was carried out in compliance with the ARRIVE guidelines. All methods were carried out in accordance with relevant guidelines and regulations. For anesthesia, a ketamine/xylazine solution was used at a dose of 130 mg/8.8 mg/kg via intraperitoneal injection. Ophthalmic ointment was placed in the eyes of animals to prevent drying. Respiration was monitored as a primary indicator of anesthetic depth. Animals were further assessed for proper levels of anesthesia by providing “toe-pinch” stimulation to the front limbs. To provide proper analgesia during a surgical procedure, the animal must reach a surgical plane of anesthesia before the procedure was started. For any observed sensitivity or pain, additional anesthesia was titered accordingly. All operative procedures were performed on a thermoregulated pad and animals were allowed to recover on a heat pad until ambulatory after tumor implantation. For pain, buprenorphine (0.05 mg/kg) was injected subcutaneously once animals were awake with a second dose administered 4–6 h later, and additional doses every 8–12 h for 48 h as needed. For implantation, tumor bits (~ 25 mm^3^) were suspended in phosphate-buffered saline (PBS) and Gentamycin (4 mg/ml) for 5 min. Mice were shaved and the site was scrubbed with betadine and 70% isopropyl alcohol. A 13-gauge sterile trocar was loaded with tumor tissue. The trocar was inserted subcutaneously to approximately the rib cage area for injection. For tumor harvest, cervical dislocation with or without anesthesia was performed. Animals were sacrificed when tumors exceeded 1 g or when animal health was compromised. These methods were consistent with the recommendations of the Panel on Euthanasia of the American Veterinary Medical Association, and were approved by the UW IACUC. Veterinary care was available 24 h a day through the UW Comparative Medicine Veterinary Services. Our animal facility was certified fully in the quality assurance program of the Department of Comparative Medicine, and was inspected regularly.

The LuCaP xenografts, each labeled by a numerical identifier, were established from samples taken from human prostate tumors during surgery or donor autopsy. Informed consent was obtained from donors whose tumor samples were used. The study was approved by a UW-Fred Hutchinson Cancer Research Center IRB. All methods employed were carried out in accordance with guidelines and regulations. Freshly harvested tumor samples (0.3–0.5 g) were minced and digested with collagenase for 2–3 h at ambient temperature with ROCK inhibitor (compound Y-27632, StemCell, Vancouver, Canada) in 3 ml culture media with gentle stirring. The cell suspension was filtered through a cell strainer and diluted with an equal volume of Hanks balanced salt solution (HBSS). The cell pellet was resuspended in HBSS and centrifuged in a Percoll discontinuous density gradient [16] to remove mouse red blood cells and fibroblasts. Cancer cells were collected at the epithelial density [epi] (ρ = 1.07) except for LuCaP 145.1 at the stromal density [strom] (ρ = 1.035) [14], and plated directly on MEF. The media was changed the next day to remove debris, and non-adhered cells. At 60–80% confluency, the cells were trypsinized and passaged on MEF; a portion of which was frozen for storage. Cell freezing in 10% DMSO/50% FBS was done in plastic straws (1/4 cc, γ-irradiated, MAI Animal Health, Elmwood, WI) with an initial gradual cooling from − 10 to − 30° at 1 deg/min followed by liquid N_2_ [13, 17]. Previously frozen LuCaP cells were thawed at 37°, rinsed in HBSS, and plated on MEF. The two LuCaP lines used in this study were adenocarcinoma LuCaP 70CR [expresses low prostate-specific antigen (PSA) and wild type AR] established from an autopsied liver metastasis, and later passaged in castrated mice to obtain the CR variant, and LuCaP 145.1 (expresses no PSA and AR) established from an autopsied liver metastasis. Without MEF support, no growth was seen.

### PENK plasmid vectors

The following four oligonucleotides were used to construct PENK vectors based on plasmids pVITRO1*neo* (neomycin/G418 resistance) and pVITRO1*bsr* (blasticidin resistance; InvivoGen, San Diego, CA): pK1EcoRV CAGGGCCCGATATCGCGTCAACTCCATGGCGCGGTTCC and pK3BamHI GCTGAGGATCCATTAAAATCTCATAAATCCTCCGTATCTTTTTTC (start and stop codons underlined) were the 5′ and 3′ primers for cloning into the *Eco*RV and *Bgl*II sites of vector mcs2; pK2BamHI GCTGAGGATCCGGCTCAACTCCATGGCGCGGTTCC and pK4AvrII TCCGAATTCCCTAGGATTAAAATCTCATAAATCCTCCGTATCTTTTTTC were the 5′ and 3′ primers for cloning into the *Bam*HI and *Avr*II sites of mcs1. The 5′ primers contained a Kozak box adjacent to the AUG codon, and the 3′ primers contained coding sequences preceding the stop codon UAA. cDNA synthesized from microdissected benign prostate tissue [18] was used for gene amplification with these PENK primers. The expected product size of full-length PENK cDNA was 800 bp. The EcoRV-BamHI cDNA and BamHI-AvrII cDNA were inserted into pVITRO1*neo* to obtain pK^2^-3*neo*, pK^2^-8*neo*; and into pVITRO1*bsr* to obtain pK^2^-1*bsr*. Each plasmid thus contained two PENK cistrons. *Pac*I digestion was used to linearize the plasmids for transfection. For comparison, the cells were transfected with a cocktail of anti-sense scTF plasmid vectors: pαLP-4*bsr*, pαSN-4*bsr*, pαPS-5*neo*, pαNL-1*neo*, where full-length scTF cDNA were inserted in the 3′ → 5′ orientation with respect to eukaryotic promoters in pVITRO1. Primer sequences with the appropriate restriction enzyme sites were synthesized for insertion of the cDNA cassettes into *Eco*RV-*Bgl*II of mcs2 and *Bam*HI-*Avr*II of mcs1. This strategy was an attempt to determine whether anti-sense transcripts could inhibit translation of endogenous scTF mRNA in LuCaP 145.1.

### Plasmid transfection of LuCaP cells

After 2 d in culture, LuCaP 145.1 cells were trypsinized, washed in HBSS, and resuspended in electroporation solution following the procedure provided by Lonza (Switzerland). The cells were electroporated in cuvettes using program S005 [14]. The plasmids (~ 1 μg) used were PENK pK^2^-3*neo* and plasmids containing the antisense constructs. Efficiency of transfection was around 10^− 4^ as reported in our previous studies [14, 19]. The cells were plated on freshly plated MEF after transfection (irradiated MEF do not replate after trypsin). At 3 d post-transfection, drug selection was started: G418 at 1 mg/ml, and blasticidin at 5 μg/ml where appropriate [19]. On drug selection, untransfected cancer cells and MEF were killed. The drug-resistant transfected cells were trypsinized after 3 d. Longer time points were not attempted since lack of continuous MEF support was expected to be deleterious. Transfected LuCaP 145.1 cells were processed for gene expression analysis. LuCaP 70CR cells were transfected by pK^2^-3*neo*. Cloning was done by picking cells of individual colonies (estimated to contain 10–50 cells) with a sterile pipetor into 6 well-plates. Outgrowth and expansion by passaging were obtained after 4 weeks or longer. Quantitation of secreted AGR2 in the culture media was carried out by AGR2 ELISA [20].

### Transfection of LNCaP cells

Prostate cancer cell line LNCaP was reprogrammed by transfection of scTF plasmids [14] to obtain neo^R^ LNCaP* (* to indicate the resultant scTF^+^B2M^lo^ cells). One clone, LNCaP*-2, was expanded in RPMI1640 media. At near confluence, the cells were harvested for transfection by pK^2^-1*bsr*, and selected for bsr^R^. Small clusters of drug-resistant cells appeared in about a week. LNCaP cells were also transfected by pK^2^-3*neo*. Photomicrographs were taken to compare cell appearance and colony morphology of LNCaP, LNCaP*, LNCaP*/PENK and LNCaP/PENK.

### Gene expression analysis

Gene expression was analyzed by RT-PCR: 94° 30 s, 57° 30 s, 72° 1 min; 35 cycles with the same oligonucleotides used in the construction of PENK plasmids. Primer pairs for B2M, LIN28A, NANOG, POU5F1, SOX2 and neo were previously reported [14]. Those for bsr were bsr5: ATGAAGACCTTCAACATCTCTCAGC and bsr3: TTAGTTCCTGGTGTACTTGAGGGG. The reaction products were detected by agarose gel electrophoresis. The expected PCR product sizes were neo 560 bp; bsr 420 bp; B2M 300 bp; PENK 800 bp; LIN28A 630 bp; NANOG 930 bp; POU5F1 1100 bp; SOX2 960 bp.

## Results

### Plasmid transfection of LuCaP 145.1 cells grown in vitro

**Fig. 1:**
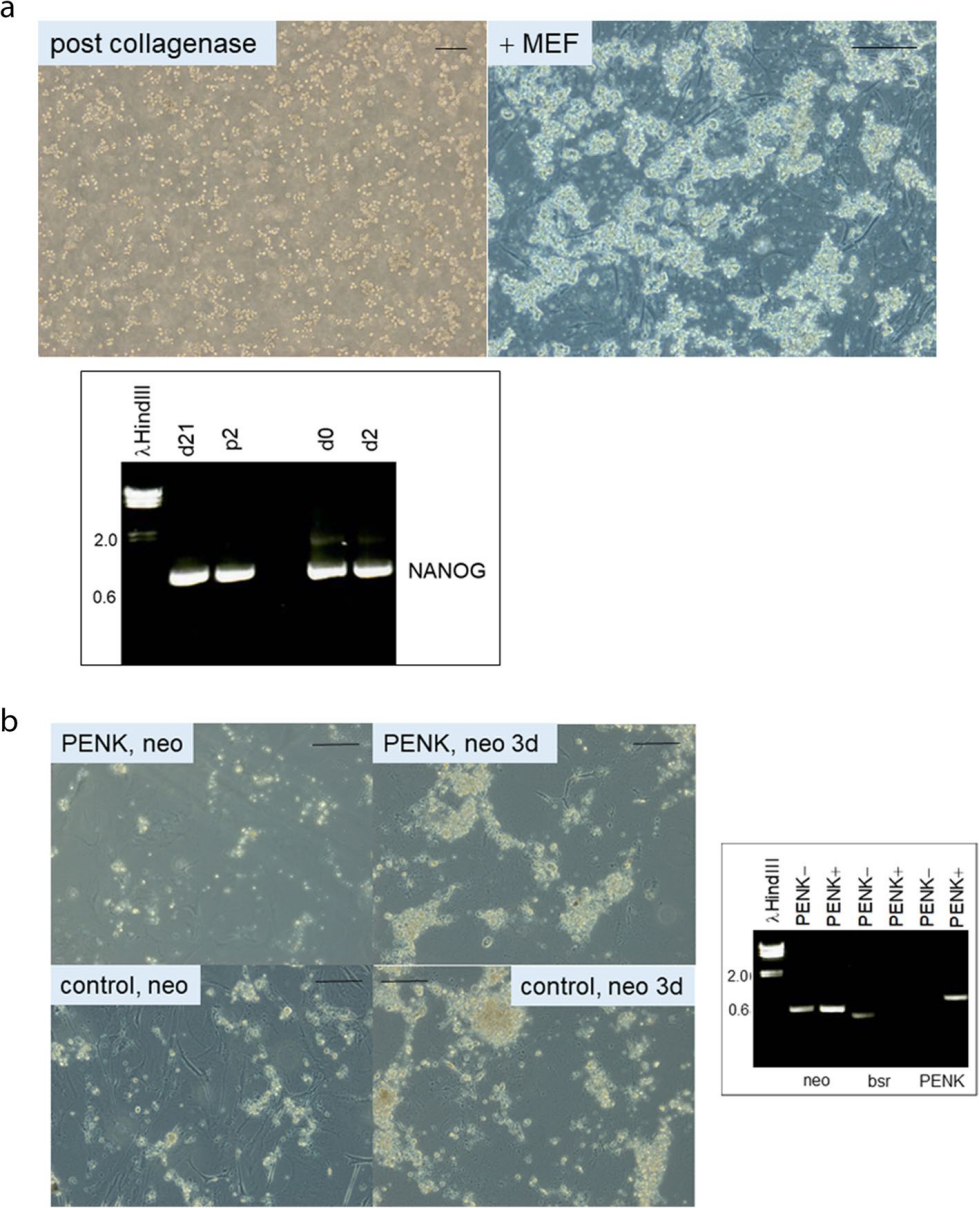
**a**. In vitro growth of small cell carcinoma LuCaP 145.1. The photomicrographs show tumor cells after collagenase digestion and plating on MEF (scale bar = 200 μm). Expression monitoring of NANOG (electropherogram) showed in vitro growth to d21, and that the cells could be passaged (p2). The signals for d0 and d2 are included. λHindIII is the DNA size marker. **b**. Plasmid transfection of LuCaP 145.1. The photomicrographs show LuCaP 145.1 cells post-transfection (PENK and α-scTF vectors) under drug selection. Drug-resistant cells proliferated for 3 d (with lysed MEF in the background) before harvest. The electropherogram confirms that PENK+ cells (LuCaP 145.1/PENK) were neo^+^bsr^−^PENK^+^ while PENK– cells (LuCaP 145.1/α-scTF) were neo^+^bsr^+^PENK^−^. The neo signal provides control for sample loading since it was expressed by both PENK+ and PENK– cells. B2M is typically used to serve as a house-keeping gene marker but in this case it was differentially expressed between PENK+ and PENK– cells

A suspension of collagenase-digested LuCaP 145.1 tumor was added to MEF. Figure 1A shows that a starter culture could be obtained with cells prepared from fresh LuCaP 145.1 tumor pieces. The top left photomicrograph shows plated LuCaP 145.1. The top right photomicrograph shows the tumor cells forming grape-like clusters attached to the underlying MEF at d1. This aggregation of LuCaP 145.1 cells precluded a clear definition of cell morphology as shown for LuCaP 70CR cells in a monolayer on MEF (see below). The gel electropherogram shows that LuCaP 145.1 cells were surviving at d21 as well as after passaging (p2) as indicated by the expression monitoring of NANOG. Also shown are d0 when LuCaP 145.1 cells were obtained after processing, and d2 when the cells were harvested by trypsin treatment for DNA transfection. No discernible changes were noted in the NANOG expression level based on PCR band intensity. Although LuCaP 145.1 cells banded at the [strom] density in Percoll, no outgrowth of mouse fibroblasts that might be present in the tumor specimen was seen (as monitored by PCR with primers for mouse B2M). PENK-expression plasmid pK^2^*neo* (containing two copies of PENK cDNA) was transfected to obtain LuCaP 145.1/PENK+. Transfection by plasmids containing anti-sense (α) constructs of the four scTF (pαPS-5*neo*, pαNL-1*neo*, pαLP-4*bsr*, pαSN-4*bsr*, where L = LIN28A, P=POU5F1, S=SOX2, N=NANOG) to obtain LuCaP 145.1/PENK–. These constructs were an attempt to inhibit scTF. However, this strategy did not work as there was no appreciable diminution of the scTF band intensity nor up-regulation of B2M (see below). Unlike anti-sense oligonucleotides, the folded full-length antisense scTF RNA could not hybridize efficiently with the endogenous sense transcripts. Instead, it afforded a suitable control for non-PENK transfection. After addition of drugs (G418 or blasticidin where appropriate), many cells including MEF were killed (Fig. 1B, left panels). Drug-resistant cells grew out at d3 as shown for LuCaP 145.1/PENK+ and LuCaP 145.1/PENK– (Fig. 1B, right panels). These cells, labeled either PENK+ or PENK–, were harvested for RT-PCR analysis at this time point to avoid the detrimental effect of long-term loss of MEF support. The electropherogram shows that the PENK+ cells were positive for neo and PENK, while the PENK– transfectants were positive for neo and bsr, and negative for PENK (Fig. 1B). These results indicated that the plasmids were integrated into the LuCaP cell genome, and the transgenes were stably expressed.

### Effect of PENK on scTF

**Fig. 2:**
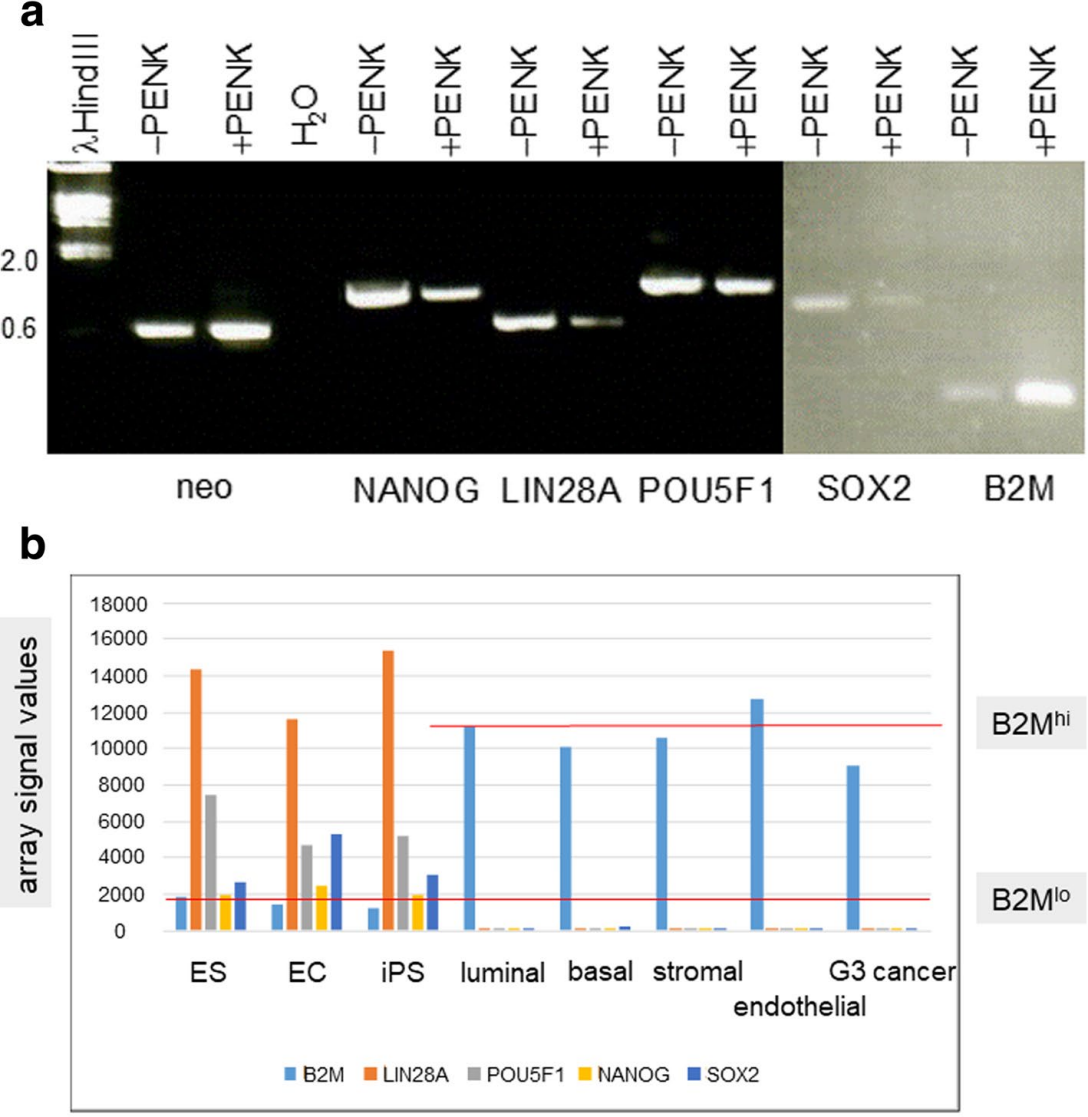
**a**. Gene expression changes induced by PENK in LuCaP 145.1. The electropherogram shows expression analysis displaying downregulation of scTF and upregulation of B2M in PENK-transfected LuCaP 145.1 (PENK+) as gauged from the intensities of the reaction products in comparison to the corresponding ones seen in α-scTF-transfected LuCaP 145.1 (PENK–). POU5F1 was not as strongly affected. The gel picture is a composite of two halves of a single run (bottom and top rows of wells with different background ethidium bromide staining). **b**. B2M levels. The histogram is generated from dataset query of transcriptomes for signal intensity values (*y*-axis) of the genes listed in the various cell types identified on the *x*-axis. The red lines highlight the B2M expression levels in stem cell types – B2M^lo^ vs. differentiated cell types – B2M^hi^

Figure 2A shows the effect of autocrine expression of PENK on LuCaP 145.1. At 3 d, PENK down-regulated the expression of scTF and up-regulated that of B2M as indicated by the difference in the PCR product band intensities. The decrease in POU5F1 was not as pronounced as that of the others since non-stem-like LuCaP lines also express this factor [13]. The neo signal could be used for sample loading control since it was present in both PENK+ and PENK–. The level of B2M was diagnostic because stem cells express 10-fold lower than differentiated cells. This fold difference was calculated from microarray probeset intensity signals for B2M in stem cell types (B2M^lo^) vs. differentiated cell types (B2M^hi^) in transcriptome datasets [21]. Stem cell types included ES cell line H1 (WA01) [22], embryonal carcinoma cell line NCCIT, reprogrammed prostate cancer-associated stromal cells [10]. Differentiated cell types included luminal, basal, stromal, endothelial [23], and Gleason pattern 3 cancer cells [18]. Thus, a phenotypic change from scTF^+^B2M^lo^ exhibited by LuCaP 145.1 to scTF^lo/−^B2M^hi^, more characteristic of differentiated cells, was produced by the forced expression of PENK. The change in B2M was a result of lowering scTF. The simultaneous changes in LIN28A, NANOG, SOX2, POU5F1 and B2M were consistent with the results obtained in stromal induction of NCCIT [7]. As a transcription regulator, PENK could apparently affect the activity of at least four stem cell genes. This effect on scTF transcription was most likely mediated by PENK protein rather than PENK mRNA. A good correspondence between mRNA and protein expression in prostate cells was found [24]. Protein expression analysis was not carried out because antibodies to NANOG and SOX2 were shown to detect these gene products in luminal-like LNCaP cells [25], which express no detectable mRNA of these scTF [9, 14]. Monoclonal antibodies to PENK were not available.

Whether scTF level would decrease to null on 7d culture was not done because a means to maintain drug-sensitive MEF under selection in culture was not available. Commercially available multi-drug resistant MEF (strain DR4) were found to be sensitive at the drug concentrations used. Attempts to transfect MEF by pVITRO1*neo* and pVITRO1*bsr* to drug resistant were unsuccessful for unknown reasons.

### Reversal of cancer cell reprogramming by PENK

**Fig. 3:**
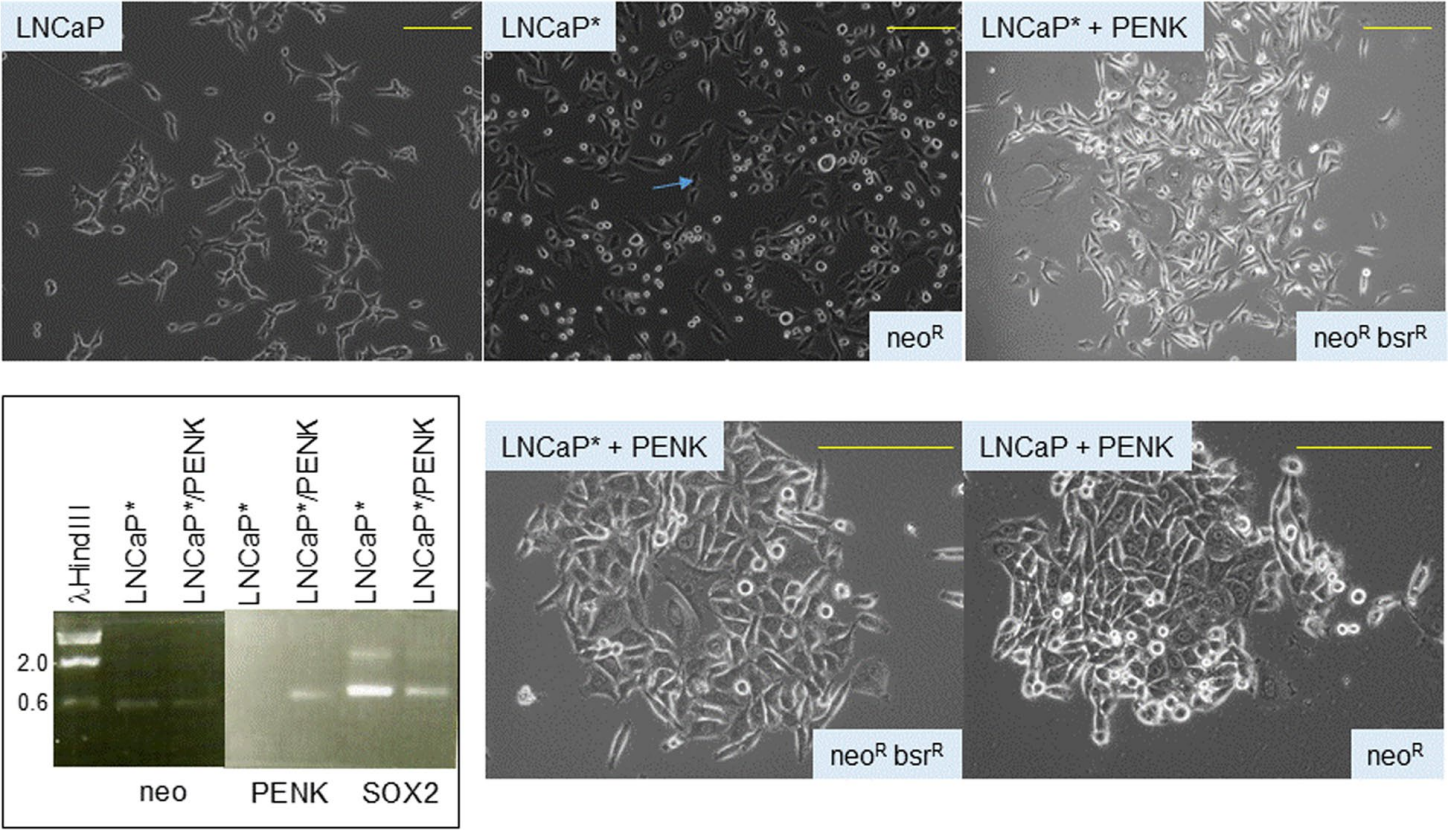
Reversal of reprogramming by PENK. The top row of photomicrographs (magnification 100x) show the cell appearance of LNCaP vs. LNCaP* vs. LNCaP*/PENK. The electropherogram confirms the downregulation of SOX2 in LNCaP*/PENK. The neo signal provides sample loading control. The gel picture is composed of two halves of a single run (bottom and top rows of wells with different background staining intensity). The bottom row of photomicrographs (magnification 200x) show a direct comparison of the colony morphology between neo^R^bsr^R^ LNCaP*/PENK and neo^R^ LNCaP/PENK

Given the influence of PENK on scTF expression in stem-like LuCaP 145.1, this factor might also be capable of reversing prostate cancer cell reprogramming? Luminal-like LNCaP cells were reprogrammed by transfection of scTF plasmids (pLP-4*neo*, pSN-2*neo*) to obtain LNCaP* showing the phenotypic change from scTF^−^B2M^hi^ to scTF^+^B2M^lo^. A selected clone LNCaP*-2neo^R^ was then transfected by pK^2^-1*bsr* (PENK plasmid with a different drug marker). After selection in blasticidin, resistant colonies were obtained. Figure 3 shows a comparison of the colony morphology of LNCaP, LNCaP*-2neo^R^ and LNCaP*/PENKneo^R^bsr^R^. A “dark” photomicrograph setting was used to highlight the “brightness” of the different cell types. Individual LNCaP cells appeared with a bright halo and were irregular in cell shape with a tendency to cluster (left photomicrograph). LNCaP*-2 cells, in contrast, appeared darker and were more regular in shape (blue arrow, middle photomicrograph). This particular appearance was similar to that of scTF-reprogrammed adenocarcinoma LuCaP 70CR, 73CR, 86.2, 92, 105CR [13]. The individual cells grew in a loose formation not in contact with the neighboring cells. LNCaP*-2/PENK appeared to regain the “bright halo” but the cell shape was different from that of LNCaP (right photomicrograph). These cells were positive for PENK, and expressed lower SOX2 than LNCaP* (with neo signal used for loading control; Fig. 3, electropherogram). Rather, the LNCaP*/PENK cells appeared similar to LNCaP transfected by PENK (LNCaP/PENK; Fig. 3). Both cell types grew in tighter clusters. Comparative analysis of the transcriptomes between LNCaP and LNCaP/PENK showed multiple gene expression changes upon PENK transfection [14]. Thus, PENK could reverse cancer de-differentiation caused by scTF reprogramming. The reversal was accompanied by cell appearance and colony morphology changes, which were similarly observed in NCCIT cells induced by stromal cell factors [7].

### Effect of PENK on adenocarcinoma LuCaP 70CR

**Fig. 4:**
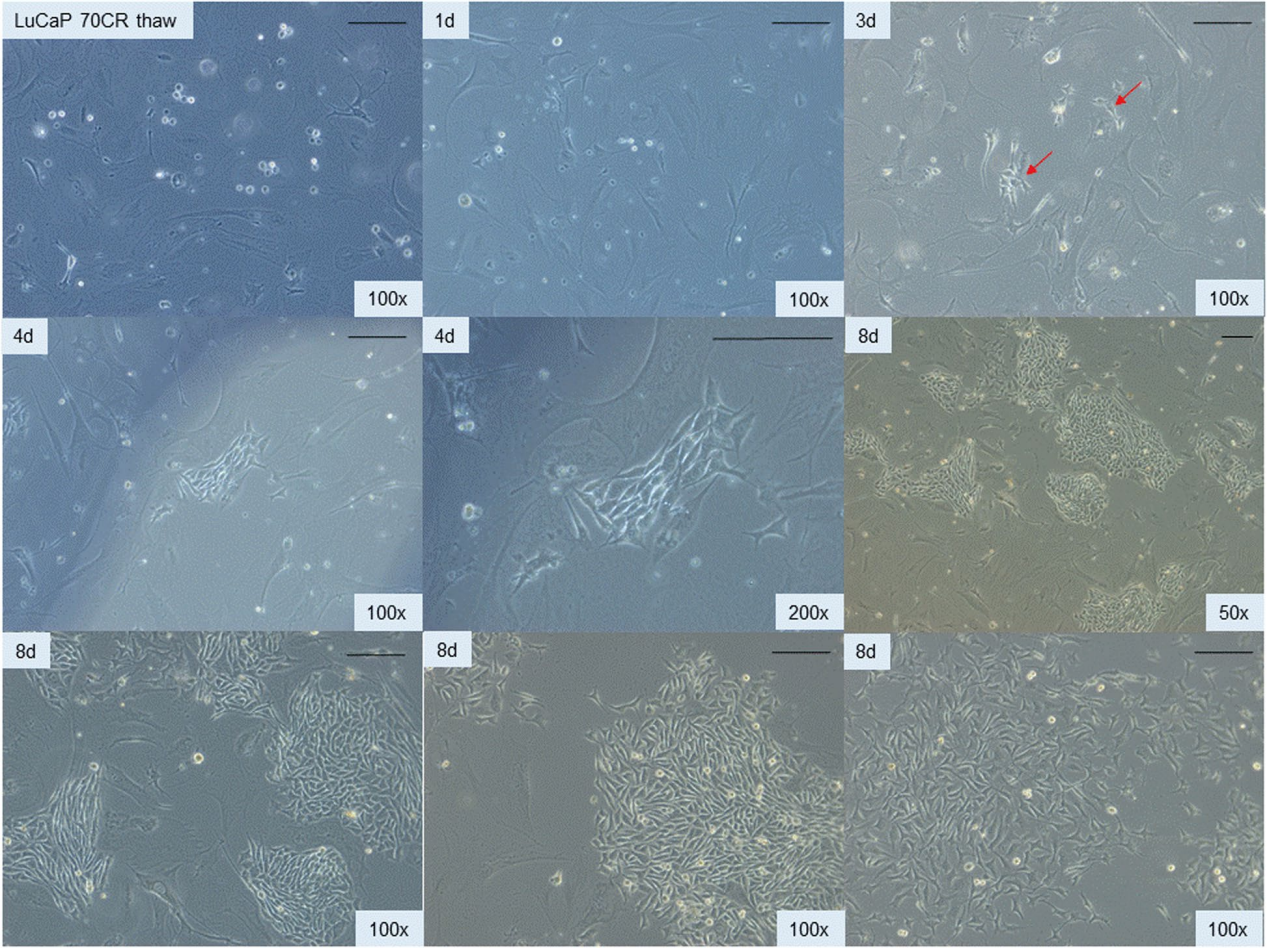
In vitro growth of adenocarcinoma LuCaP 70CR. These photomicrographs (magnification indicated) show the expansion of LuCaP 70CR from thaw to d8 in culture with MEF. Small cell clusters could be observed at d3 (top right, red arrows). At d8, two colony morphologies were seen (compare bottom middle and right)

Given the effect of PENK on LNCaP, PENK could also affect non-stem-like LuCaP cells. Luminal-like scTF^−^B2M^hi^ LuCaP 70CR was transfected by pK^2^-3*neo*. LuCaP 70CR cells were previously adapted to in vitro culture and were frozen for long-term storage [13]. Figure 4 shows in vitro culture of LuCaP 70CR on MEF after thawing. At d3, small clusters of epithelioid cells were detectable (red arrows). These individual small colonies expanded that by d8 large proliferating colonies were evident. Most of the colonies showed a compact morphology, while a few showed a “looser” morphology (bottom right panel). The epithelioid appearance of these cells distinguished them from the underlying mouse feeder fibroblasts. The tumor cells could be passaged by trypsin treatment, and replating on a freshly plated irradiated MEF. This result demonstrated that in vivo-passaged LuCaP cells could be frozen for long-term storage, and thawed for continuous culture with MEF in serum-supplemented media. The thawed LuCaP 70CR cells survived cloning and multiple passages in the course of over 2 months.

**Fig. 5:**
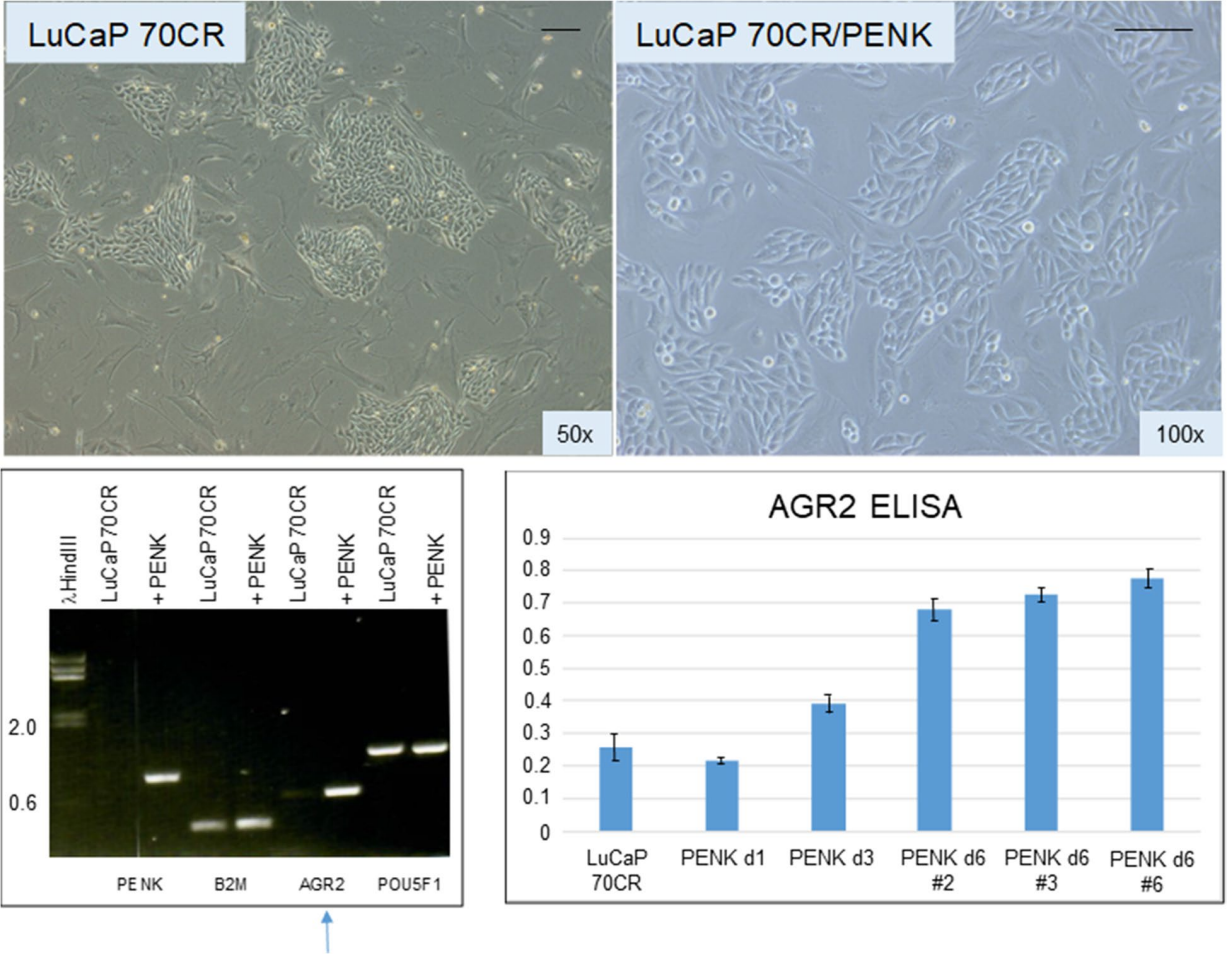
PENK transfection of LuCaP 70CR. The photomicrographs show LuCaP 70CR before and after PENK transfection. The electropherogram shows an increase in the expression of AGR2 mRNA (blue arrow). Increased AGR2 expression was validated by measurement of secreted AGR2 in the culture media. The histogram is a representation of the optical density values (*y*-axis) from ELISA measurement. PENK d6 #2, 3, and 6 are three picked LuCaP 70CR/PENK cell clones analyzed after 6 d in culture

Fig. 5 shows LuCaP 70CR plated on MEF before and after PENK transfection. Unlike for LuCaP 145.1, PENK did not affect the expression of B2M and POU5F1 (the other three scTF are not expressed by this line). Of note, PENK signaling increased expression of the adenocarcinoma antigen AGR2 (Fig. 5, electropherogram). The increase was confirmed by ELISA of secreted AGR2 in the culture media of three cloned LuCaP 70CR/PENK cells (Fig. 5, histogram). The elevated AGR2 expression was indicative of cancer cell differentiation induced by PENK in adenocarcinoma cells.

## Discussion

Cellular differentiation entails inactivation of scTF. Many ongoing research efforts in regenerative medicine are undertaken to identify specific agents, either produced naturally in our body or chemical substitutes, to induce differentiation of stem or progenitor cells into functional cell types of cardiomyocytes, kidney cells, for example. Prostate stromal PENK was identified as a possible prostate-specific signaling molecule. Using small cell carcinoma LuCaP 145.1 as a stem-like cell type, we showed that PENK could induce these cancer cells to undergo differentiation with down-regulation of scTF and simultaneously up-regulation of B2M. Although LuCaP 145.1 cells contain accumulated DNA mutations [26], they retain responsiveness to a stromal inductive factor. Complementary to this result, PENK could reverse cancer de-differentiation from luminal-like adenocarcinoma to stem-like small cell carcinoma induced by scTF. The sequence of scTF^−^B2M^hi^ LNCaP cells → scTF^+^B2M^lo^ LNCaP* → scTF^−^B2M^hi^ LNCaP*/PENK was trackable by changes in colony morphology and cell appearance. PENK can inhibit reprogramming by preventing activation of the scTF genes. We observed this inhibition in our reprogramming of prostate stromal cells, in which we were able to obtain stem-like cells from PENK-negative cancer-associated stromal cells but not PENK-positive benign tissue stromal cells [10]. Thus, if prostate cancer de-differentiation can be reversed (by the action of stromal factors), then the disease can be better managed to prevent progression. Cancer differentiation therapy as shown first by retinoic acid on promyelocytic leukemia is a well-recognized treatment strategy [27]. We suggest that PENK could likewise be effective in promoting cancer differentiation of a solid tumor. As PENK is a natural product of our body, its clinical application would be less harmful than, say, radiation or chemodrugs. With further research, it is possible that PENK plus other stromal factors could induce cancer cells to a pseudo-normal state (as shown by our experiments with stromal induction of NCCIT). That cancer cells can undergo de-differentiation and differentiation means that mutations in the cancer cell genome and aneuploidy would not pose a problem in the application of this therapy.

Small cell carcinoma, although rare (as would be expected on the need to sequentially re-activate multiple scTF), are found in many solid tumors: 15% lung cancer, < 1–5% prostate, bladder, pancreatic, breast, ovarian. More recently, nearly 20% prostate cancer patients treated by new anti-androgen therapies [28] were shown to harbor small cell carcinoma. Our finding of scTF^+^B2M^lo^ in small cell carcinoma LuCaP 145.1 suggests that this phenotype could characterize small cell carcinoma in general. For example, although the lung equivalent of PENK has not been identified, PENK perhaps could also induce lung small cell carcinoma to differentiate by down-regulating scTF.

The up-regulation of AGR2 in LuCaP 70CR showed that PENK could also affect gene expression of adenocarcinoma cells. This contrasts with the down-regulation of AGR2 in reprogrammed LuCaP 70CR* [13]. Small cell carcinoma does not express AGR2 [5]. AGR2 is associated with prostate cancer differentiation as Gleason 3 (well-differentiated) cancer cells show a 10-fold higher level than Gleason 4 (less differentiated) cancer cells [4]. Patients whose tumors with high AGR2 expression have a 9-fold survival advantage than those whose tumors with low AGR2 expression [29]. An association between AGR2 and differentiation is also found in breast cancer, where better survival is linked to AGR2 [30]. More importantly, AGR2 is a unique tumor-associated antigen because cancer cells express the extracellular form eAGR2 (on the cell surface and secreted) while normal cells express the intracellular form iAGR2 (localized to the endoplasmic reticulum) [15, 31]. Therefore, antibodies raised against AGR2 would recognize specifically cancer cells and not AGR2-expressing normal cells. If PENK can prevent cancer cell de-differentiation and maintain high eAGR2 expression, then anti-AGR2 immunotherapy [19] would be a potentially effective therapy.

**Fig. 6:**
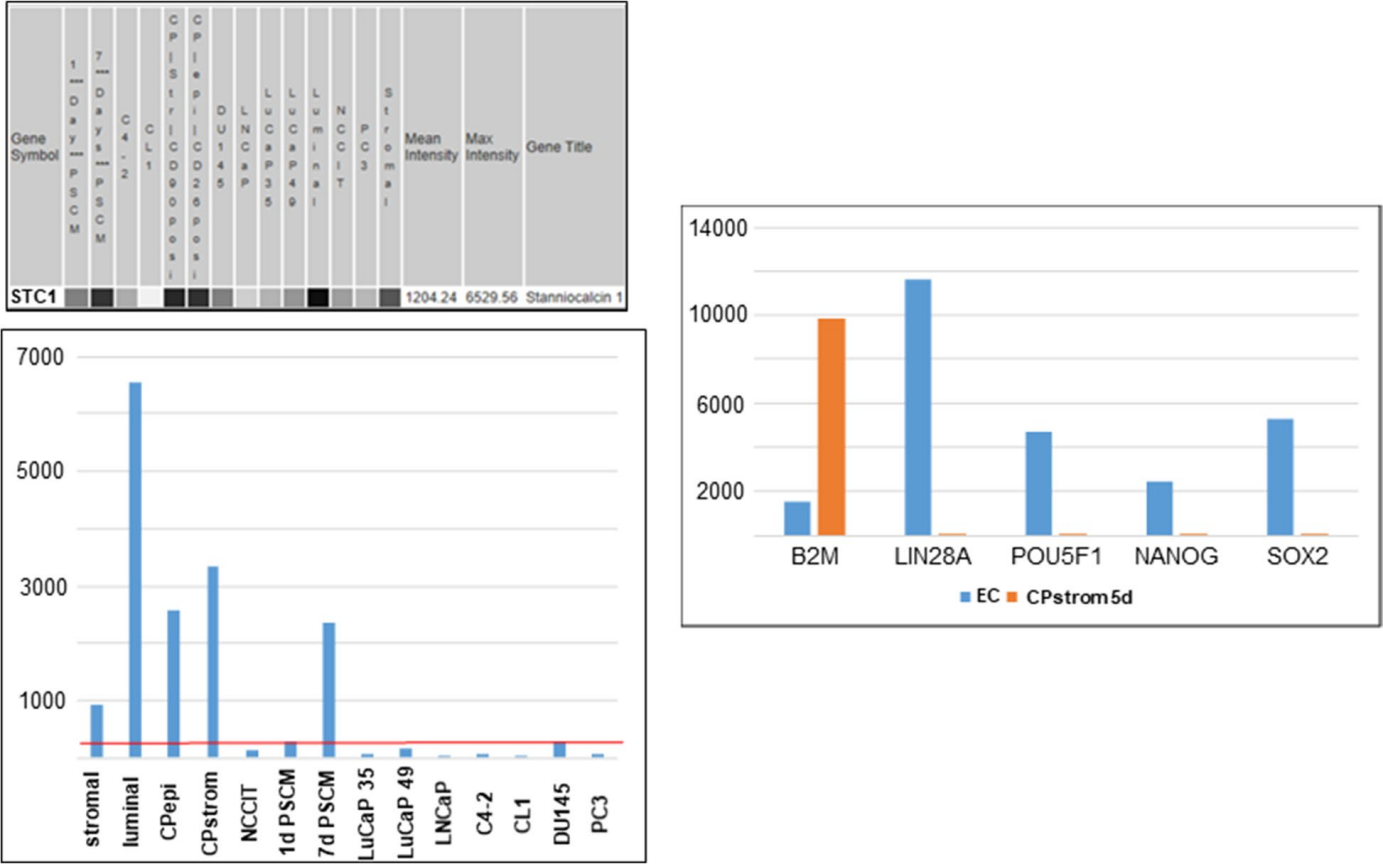
Left panels. STC1 expression pattern. Array signal intensity values (on a gray scale) were retrieved from transcriptome datasets (top) and displayed in a histogram format (bottom). The intensity values were retrieved from Affymetrix microarray datasets archived in our SCGAP Urologic Epithelial Stem Cells Project. The values are the average after clicking coalesce replicates and probesets. Dataset query using this public database is described in ref. 21. The red line shows the low expression of STC1 in cancer cell lines and xenografts. Right panel. Cancer-associated stromal induction of NCCIT. Down-regulation of scTF and up-regulation of B2M can be seen with induction by cancer-associated (CP) stromal cell conditioned media. Unlike normal prostate stromal cells, these CP stromal cells lack expression of PENK but not STC1

Full differentiation from stem-like cancer cells to luminal-like depends likely on more than one factor. Other identified stromal factors like stanniocalcin 1 (STC1) [2] may play a role. STC1, like PENK [32], is known to be involved in early development [33, 34]. In the NCCIT experiment, STC1 was induced at an earlier time point than PENK [7]. Although identified as stromal, STC1 is also expressed by the epithelial cells, well-differentiated cancer cells and cancer-associated stromal cells. STC1 expression is, however, much reduced in prostate cancer cell lines and xenografts (Fig. 6A). Conditioned media of STC1^+^PENK^−^ cancer-associated stromal cells (isolated from a Gleason 3 tumor) were still able to induce NCCIT to down-regulate scTF and up-regulate B2M (Fig. 6B) but without induction of PENK [7]. Gleason 3 cancer cells show a degree of differentiation (scTF^−^B2M^hi^, Fig. 2B) in the absence of PENK. It is possible that STC1 and PENK could act in concert towards differentiation, with additional contribution from many other differentially expressed candidate genes between CD49a^+^ prostate and CD13^+^ bladder stromal cells [2], between CD49a^+^ prostate stromal and CD90^+^ cancer-associated stromal cells [11]. The successful adaptation of PDX lines to in vitro growth makes possible to study in depth the molecular mechanism of stromal-epithelial interaction in cancer such as co-culture with prostate vs. bladder, prostate cancer-associated stromal cells isolated from different Gleason grades.

**Fig. 7:**
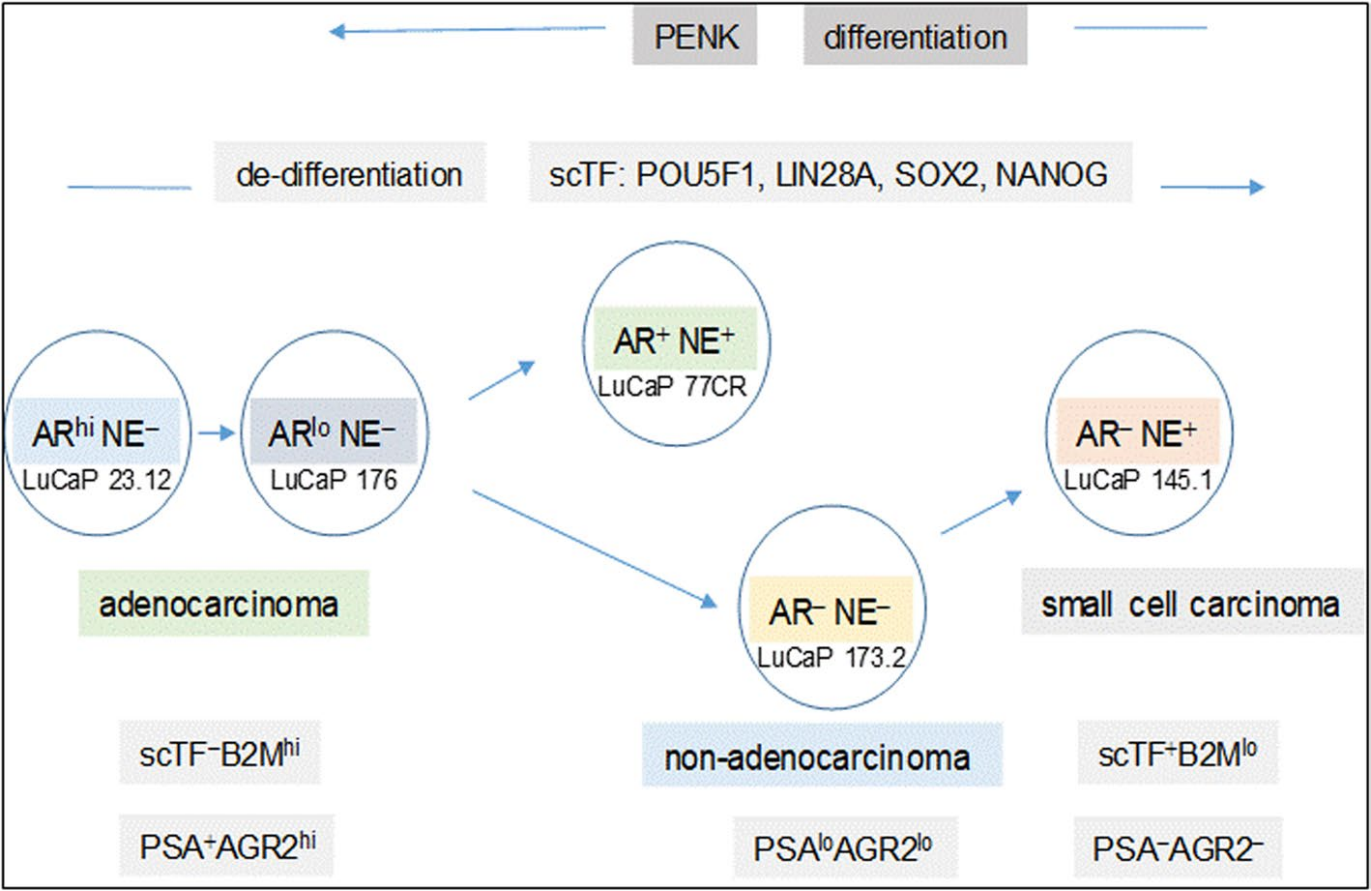
Lineage of prostate cancer cells. In this schematic, the different prostate cancer cell types are identified by AR and NE expression. The progression from AR^+^NE^−^ luminal-like to AR^−^NE^+^ stem-like is through the activation of scTF, which is equivalent to de-differentiation. Stem-like cancer cells respond to stromal factors such as PENK by undergoing differentiation changing from a scTF^+^B2M^lo^ phenotype to that of scTF^−^B2M^hi^. The cell types are represented by different LuCaP lines. The AR^+^NE^+^ and AR^−^NE^−^ types represent an intermediate that can become AR^−^NE^+^ from losing the AR expression program and gaining the NE program by the former, and gaining the NE program by the latter

Using AR expression to denote luminal-like adenocarcinoma and neuroendocrine (NE) expression to denote stem-like small cell carcinoma, prostate cancer differentiation (from NE^+^ stem-like to AR^+^ luminal-like) and de-differentiation (from AR^+^ luminal-like to NE^+^ stem-like) can describe a proposed lineage relationship among the different cancer cell types (Fig. 7). Luminal expression is governed by AR signaling, while NE expression in stem-like is due to SOX2 because this scTF alone can reprogram human fibroblasts into multipotent neuronal stem cells, which can then be induced to differentiate into mature neuronal cell types [35]. The LNCaP experiment supports the validity of this model of bi-directional changes: LNCaP → LNCaP* → LNCaP*/PENK ≅ LNCaP/PENK. In the LuCaP series [36], the AR^hi^NE^−^ type is represented by LuCaP 23.12 (35, 96CR and many others), AR^lo^NE^−^ by LuCaP 176 (and others), AR^−^NE^+^ by LuCaP 145.1 (93, 145.2), AR^−^NE^−^ by LuCaP 173.2 (with squamous features, possibly activated by non-AR, non-NE signaling), AR^+^NE^+^ by LuCaP 77CR (Fig. 7). Note although expression of AGR2 appears linked to that of AR, it is not strictly. Cancer cells in local spread show low AGR2 expression [5] as exemplified by luminal-like LNCaP and LuCaP 35. In contrasts, cancer cells in distal spread show high expression except small cell carcinoma. Variants derived from selection of LNCaP in androgen-depleted media show high AGR2 expression [20].

## Conclusions

Loss of prostate cancer differentiation involves the activation of scTF. Activity of these factors convert scTF^−^B2M^hi^ luminal-like adenocarcinoma to scTF^+^B2M^lo^ small cell carcinoma. Stromal PENK can counteract scTF to induce differentiation, characterized by down-regulation of scTF and up-regulation of B2M. Up-regulation of AGR2 is another characteristic of differentiation upon introduction of PENK into adenocarcinoma cells. The multiple mutations and aneuploidy in cancer cells appear not to inhibit differentiation or de-differentiation.

## Supplementary Information

**Additional file 1.**

## Data Availability

Data availability is not applicable as there are no mandated raw data types for deposition. The generated cell lines are available through Materials Transfer agreement with UW.

## Abbreviations

AGR2: Anterior gradient 2
AR: Androgen receptor
B2M: β2-microglobulin
CD: Cluster designation cell surface antigens
CPstrom: Prostate cancer-associated stroma
CR: Castration resistant
EC: Embryonal carcinoma (cell line NCCIT)
[epi]: Epithelial cell density
ES: Embryonic stem
FBS: Fetal bovine serum
HBSS: Hanks balanced salt solution
iPS: Induced pluripotent stem
MEF: Mouse embryonic fibroblast
NE: Neuroendocrine
NPstrom: Benign prostate stroma
PDX LuCaP: Patient-derived xenograft lines
PENK: Proenkephalin
PSA: Prostate-specific antigen
RT-PCR: Reverse transcriptase-polymerase chain reaction
scTF: Stem cell transcription factors LIN28A, NANOG, POU5F1, SOX2
STC1: Stanniocalcin 1
[strom]: Stromal cell density
