# Supplementary figures and images for: The opposing action of stromal cell proenkephalin and stem cell transcription factors in prostate cancer differentiation
Source: BMC Cancer. 2021 Dec 15;21:1335. doi: 10.1186/s12885-021-09090-y (PMC8675470; doi:10.1186/s12885-021-09090-y)

## Slide 1
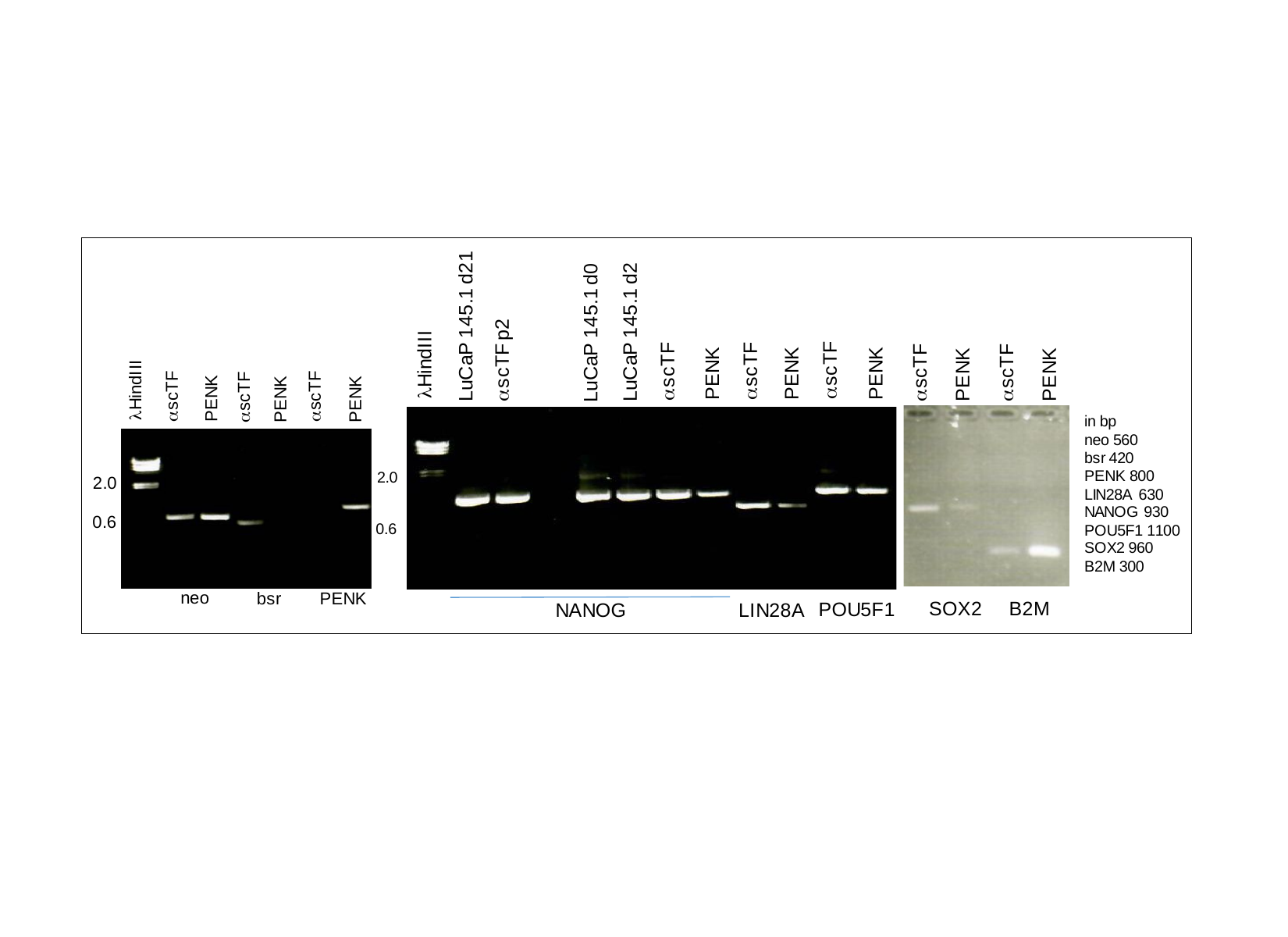

Supplement: Supplementary file 1 — Additional file 1. [file 12885_2021_9090_MOESM1_ESM.pptx]
